# First case of *Mycobacterium marseillense* lymphadenitis in a child

**DOI:** 10.1186/s13052-017-0413-5

**Published:** 2017-10-10

**Authors:** A. Azzali, C. Montagnani, M. T. Simonetti, G. Spinelli, M. de Martino, L. Galli

**Affiliations:** 1 Department of Health Sciences, University of Florence, Anna Meyer Children’s University Hospital, Viale Pieraccini, 24, I-50139 Florence, Italy; 2 Pediatric Infectious Diseases Unit, Anna Meyer Children’s University Hospital, Florence, Italy; 3 Tuscany Regional Reference Centre for Mycobacteria, Microbiology and Virology Unit, Careggi Hospital, Florence, Italy; 4 Maxillo-Facial Surgery Unit, Neurosensorial Department, Azienda Ospedaliero-Universitaria Careggi, Florence, Italy

**Keywords:** Lymphadenitis, Children, Mycobacterium marseillense, Nontuberculous mycobacteria

## Abstract

**Background:**

Nontuberculous mycobacteria (NTM) are pathogens that commonly affect the paediatric population and its most frequent manifestation is a cervicofacial lymphadenopathy. With the improvement of technologies, new species have been recently identified.

**Case presentation:**

We report the first case of NMT lymphadenitis in a child caused by *Mycobacterium marseillense,* a newly described species belonging to *Mycobacterium avium complex*.

**Conclusions:**

Improving the identification of these newly discovered mycobacteria, further information will be available about their clinical involvement and their best treatment.

## Background

Nontuberculous mycobacteria (NTM) are pathogens causing different patterns of diseases, ranging from an asymptomatic colonization to a widespread dissemination, depending on the host immune competence [1]. NMT can be found in soil, water, milk, eggs, vegetables and animals. In immunocompetent children, they usually cause an unilateral, painless, progressive lymphadenopathy that lacks constitutional symptoms. Submandibular and cervical lymph node stations are mainly involved, while the incidence of preauricolar, inguinal and axillary area affection is lower [2].

Cervicofacial NTM lymphadenitis affects mainly children below 4 years of age, while it is rarely diagnosed in adolescents. The estimated incidence of NTM disease is of 0.6 to 1.6 cases per 100,000 children per year and it increases during spring and late winter [2].

More than 150 species of NTM have been recognized, but less than 20 species have been identified in human infections [3]. Members of *Mycobacterium avium complex* (MAC) are the causative agents of more than 75% of cases of NTM lymphadenitis, followed by *Mycobacterium haemophilum* (6%) [4].

*Mycobacterium marseillense* has recently been described as a new species belonging to the MAC complex [5]. We report on the first case of NMT lymphadenitis caused by *Mycobacterium marseillense* in a child.

## Case presentation

**Fig. 1:**
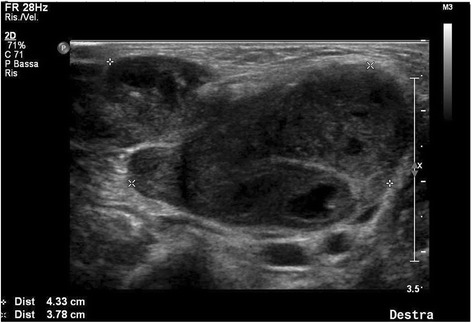
Preoperative ultrasound of patient’s lymph node

We evaluated a 4 years old girl, previously healthy, with no relevant family medical history. She was conducted to our clinic because of a submandibular lymphadenitis. A month before, during a 3 days fever period, a painful submandibular swelling was noted. Neck ultrasound (US) showed a 2-cm diameter lymphadenopathy with a conserved structure. Therapy with ibuprofen was then suggested. Because of symptoms persistence, a control US was repeated after 5 days. The lymph node was enlarged and presented a subverted structure, with hypoechogenic areas and an absent roundness index (Fig. 1). Therapy with co-amoxiclav was immediately started at a dosage of 80 mg/kg/day for 6 days. Blood tests showed a slight increase in C-reactive protein values (53 mg/L; normal value <50 mg/L) and erythrocyte sedimentation rate (31 mm/h); serology for Cytomegalovirus confirmed a previous infection, while IgM for Epstein Barr Virus (EBV) were positive. Blood smear was normal. Tuberculin skin test resulted positive (10 mm infiltration diameter). QuantiFERON-TB Gold In-tube test and chest X-ray resulted negative.

Neck US performed after 20 days was unchanged. Therefore the girl was referred to our infectious disease unit for further investigations. At physical examination, a fixed right submandibular lymphadenopathy, with an elastic consistency, painless and with no superficial skin alterations was detected. No other significant clinical features were described. Blood tests showed no alteration in blood count, inflammatory markers, liver function, lactic dehydrogenase, coagulation. *Francisella tularensis* and *Toxoplasma gondii* serology were negative. EBV serology showed evidence of a past infection.

In the suspicion of an NTM lymphadenitis, antimicrobial therapy with clarithromycin (15 mg/kg/die), and rifampicin (20 mg/kg per day) was started. A magnetic resonance imaging showed a confluent lymphonodal pack inside the inferior pole of the right parotid gland (4 × 4.2 × 2.9 cm) that was enhanced after contrast medium infusion. The enhancement involved also the whole parotid gland parenchyma and the ipsilateral sternocleidomastoid muscle.

Due to sudden enlargement of swelling, exeresis of the lymph node was performed. Bacterioscopic exam showed a positivity for acid-fast bacilli, and the histological examination evidenced a granulomatous inflammation. Culture from the biopsy grew a *M. marseillense* strain, identified using a commercial kit (Hain Genotype Mycobacteria CM, Hain Lifescience, Germany) and sequencing the spacer region interposed between 16S and 23S rRNA genes, as described previously [6]. Because of a slow healing up of the surgical scar, ethambutol (25 mg/kg/die) was added to the previous therapy. Antimicrobial treatment was well tolerated by the patient and no adverse effect were reported. Blood exams and clinical assessment were periodically performed in order to evaluate potential drug-induced toxicity. Therapy was finally stopped 2 months after surgery.

Periodical blood tests and neck US were performed. At 10 months follow up visit, no relapse was found and the surgical scar was completely healed.

## Discussion and conclusion

To the best of our knowledge, this is the first reported case of lymphadenitis due to *M. marseillense* in a child, moreover immunocompetent. NTM infection should always be considered in the differential diagnosis of chronic lymphadenopathies, especially in children below 4 years of age. MAC is the most common cause of NTM lymphadenitis, with *M. avium* being the species most frequently isolated. However, mycobacterial taxonomy is often a challenge for microbiologists, because of the new species that are continuously described due to the application of molecular techniques [3].

*M. marseillense* was firstly described in 2009. It is a slowly growing, small, acid-fast bacillus, belonging to the MAC complex, showing the closest similarity with *Mycobacterium chimaera* [5]*.*

Clinical features of *M. marseillense* infections are not yet clearly known due to the extreme rarity of these conditions. Up to now, only two cases of pulmonary infection and two cases of isolation in the *sputum* of patients with cystic fibrosis have been described [7]. One report was about a 56 years old woman with systemic lupus erythematosus who presented a story of cough and purulent sputum with chest consolidation of the right middle lobe at computed tomography (CT) scan, unresponsive to multiple courses of antibiotics. After bronchoscopy, *M. marselleinse* was isolated in bronchoalveolar lavage [8]. The other report was about a 65 years old immunocompetent man who presented at chest radiographs a diffuse nodular opacity and bronchial thickening, confirmed by high-resolution CT. On bronchoalveolar lavage, a nontuberculous *Mycobacterium* strain was isolated by culture and preliminarily identified as *M. intracellulare*. Rifampin, isoniazid and amikacin were initially prescribed, with clinical improvement. In the subsequent 4 years, the patient presented intermittently positive culture despite the various therapeutic cycles. The last drug regimen was with ethambutol, rifampin and azithromycin. Anyway, cough and sputum production persisted despite negative microbiological test results. Moreover, radiological follow up evidenced a progressive pulmonary involvement [9].

As no other cases of lymphadenopathy due to *M. marseillense* were described, no specific indications for the treatment of this kind of infection were present in the literature. Excision was performed as indicated in NTM lymphadenitis and allowed the identification of the germ. Therapy for NTM infections with macrolide and rifampin was given as suggested by American Thoracic Society and the Infectious Diseases Society of America guidelines [10], and ethambutol was later added due to delay in the healing of the surgical scar. Clinical and radiological follow up showed a decrease of lymph node dimension with an almost complete recovery at 6 months.

*M. marseillense* role in lymphadenitis could be underestimated given the similarities to *M. intracellulare.* Further studies are needed to understand the clinical involvement of these newly discovered mycobacteria in NTM lymphadenitis and their best treatment.

## Abbreviations

EBV: Epstein Barr Virus
*M. marseillense*: *Mycobacterium marseillense*
MAC: *Mycobacterium avium complex*
NTM: Nontuberculous mycobacteria
